# Surgical management of primary and idiopathic internal hydrocephalus in dogs and cats

**DOI:** 10.3389/fvets.2024.1435982

**Published:** 2024-07-04

**Authors:** Martin J. Schmidt, Daniela Farke

**Affiliations:** Department of Veterinary Clinical Sciences, Neurosurgery, Neuroradiology and Clinical Neurology, Small Animal Clinic, Justus-Liebig-University, Giessen, Germany

**Keywords:** canine, feline, hydrocephalus, magnetic resonance imaging, VPS

## Abstract

Ventriculoperitoneal shunt placement is an effective method to treat internal hydrocephalus in dogs and cats. Although it has a long history in veterinary medicine, the technique continues to evolve. Despite continuing attempts to reduce the incidence of associated complications, shunt failure remains a major problem, and often leads to multiple hospital admissions. This review gives an overview about current knowledge of ventriculoperitoneal shunting techniques in animals, applicable shunt hardware as well as shunt-associated complications and their prevention and treatment.

## Introduction

Congenital internal hydrocephalus is a common malformation in dogs and cats (1–3). However, internal hydrocephalus is heterogeneous in nature and can be due to a wide spectrum of other pathological entities (4). Underlying causes include primary brain malformations (cysts, aqueductal stenosis, interventricular septae etc.), as well as secondary causes, including intraventricular hemorrhage, intracranial tumors, and infectious diseases. In many cases, the underlying cause remains undetermined (idiopathic hydrocephalus) (4). Untreated hydrocephalus can lead to many clinical signs including ataxia, blindness, behavioral abnormalities, and vestibular signs, and can also lead to death (5). Ventriculoperitoneal shunting (VPS) is currently the best treatment option (3, 6). Whereas VPS is one of the most common neurosurgical procedures in humans (7), there is less experience with this technique in hydrocephalic animals. Despite perfect shunt placement and function, short-term and long-term complications may prevent improvement of clinical signs and survival of the animals (8–10). Thorough preoperative planning, selection of appropriate hardware, and postoperative monitoring can help to reduce the occurrence of complications and the need for shunt revision. In this review, we summarize current knowledge on VPS in dogs and cats. The most common complications of VPS as well as strategies for prevention and their treatment are discussed.

## Review

### Surgical technique and equipment

The purpose of VPS is to divert excess cerebrospinal fluid (CSF) from the cerebral ventricles to the abdominal cavity. Although all available shunt systems have the same basic components in order to achieve this, they differ in a few details. Our experience is mostly confined to one particular shunt system.

**Figure 1:**
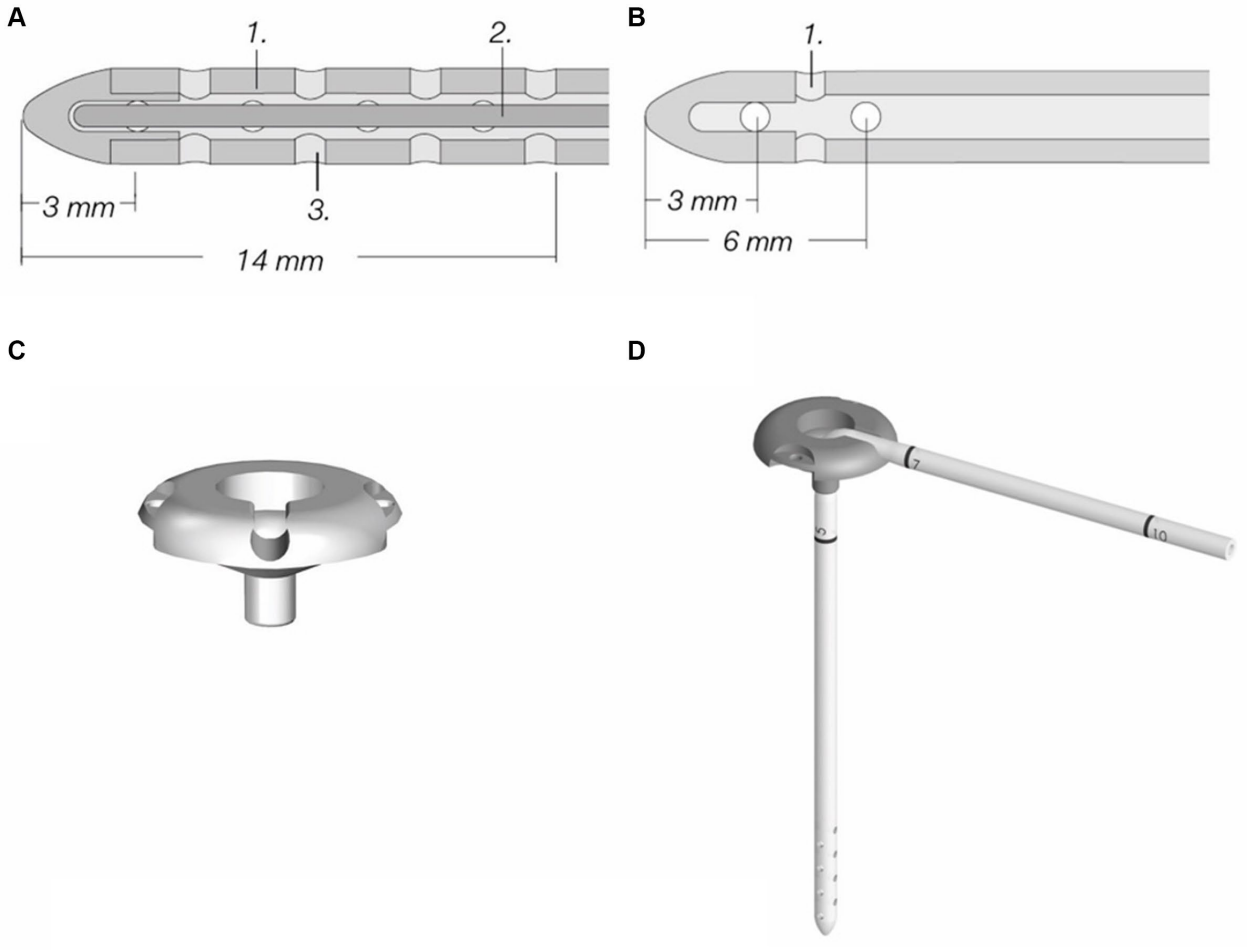
Components of a ventriculoperitoneal shunt system (MIETHKE GmbH & Co. KG, Potsdam, Germany). **(A)** Standard ventricular catheter with mandrel, containing eight drainage segments; inner diameter is 1.2 mm, outer diameter is 2.5 mm. **(B)** Thomale catheter with only three drainage segments, inner and outer diameters are the same as in standard catheters. **(C)** Shunt deflector. **(D)** Shunt deflector with ventricular catheter. Permission granted by Christoph Miethke GmbH & Co. KG, Potsdam, Germany; www.miethke.com.

The ventricular catheter is a flexible silicone tube that is placed in the lateral ventricle. It contains a mandrel that helps to avoid kinking of the catheter during implantation through the cerebral cortex. The ventricular catheter contains regular holes arranged in several drainage segments, each containing four holes that take up CSF (Figure 1A). The distance from the tip is marked on the catheter allowing for setting of the appropriate insertion depth. A smaller number of drainage segments in a short distance to the catheter tip (Thomale catheter, Figure 1B) is used in humans in order to avoid potential parenchymal contact with the drainage segments (see below) (11). This ventricular catheter type is especially useful for smaller dogs and cats with smaller ventricular dimensions.

**Figure 2:**
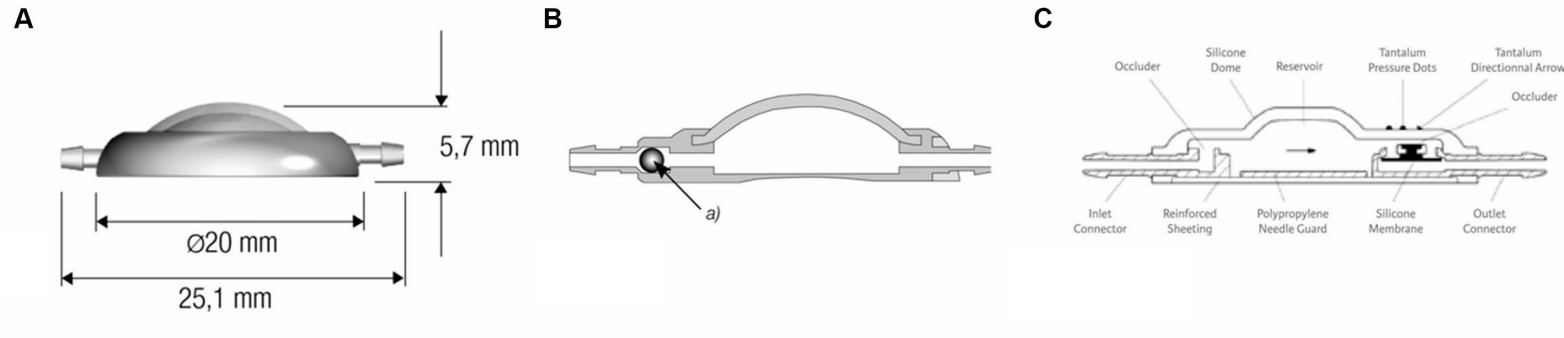
**(A)** Dome reservoir with titanium base and silicone dome, lateral view. **(B)** Control reservoir with non-return valve, technical drawing sagittal section (MIETHKE GmbH & Co. KG, Potsdam, Germany). Reprinted with permission Christoph Miethke GmbH & Co. KG; www.miethke.com. Technical drawing of the Contour-Flex^™^ Valve System (Integra Neuroscience) incorporating an internal diaphragma valve, which is mounted distal to an integral pumping reservoir.

The ventricular catheter has variable types of shunt deflectors, allowing 90° deflection of the ventricular catheter in the burr hole without kinking the catheter (Figures 1C,D). These are adjustable on the catheter and help to pre-set the maximum depth of the ventricular catheter. The shunt systems usually incorporate a reservoir consisting of a solid base and a silicone dome (Figure 2A). Punctuating the dome allows sampling of CSF out of the shunt tube. Some reservoirs include a non-return valve in the proximal inlet connector that avoids flow in the direction of the proximal catheter during the pumping procedure (control reservoir; Figure 2B). Pressing the dome results in a CSF pressure wave towards the valve, which is a common site of obstruction in humans. A reservoir can be a separate part of the shunt, but can also be combined with a valve (Figure 2C).

**Figure 3:**
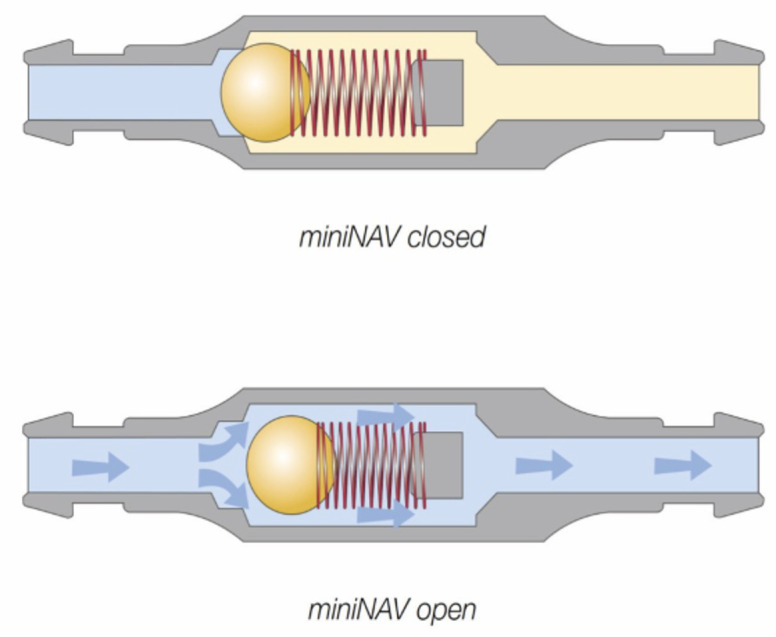
Differential pressure valve containing a ball in cone and spring valves (paediGAV^®^, MIETHKE GmbH & Co. KG, Potsdam, Germany). The titanium housing contains a spring fixed on a ball that blocks the passage. The resistance of the spring defines the opening pressure. If the intraventricular pressure exceeds the valve opening pressure, the spring force that otherwise holds the ball-cone valve closed is overcome. The spring is compressed, and the ball moves out of the cone, opening up a gap for fluid drainage. Permission granted by Christoph Miethke GmbH & Co. KG; www.miethke.com.

An important component of the shunt system is a valve that regulates the driving pressure through the shunt. To avoid overshunting (see below), the shunt systems contain valves that act like on-off switches, opening when the intraventricular pressure (IVP) exceeds the valve’s opening pressure, allowing egress of CSF until IVP falls below the opening pressure (differential pressure valve system). Available valves either use a ball-in-cone and spring mechanism, in which the resistance of the spring reflects the opening pressure or involves a membrane that closes the outlet of the valve, but gradually deforms in response to increasing pressure (diaphragma valve, codman Medtronic) (12) (Figure 3).

**Figure 4:**
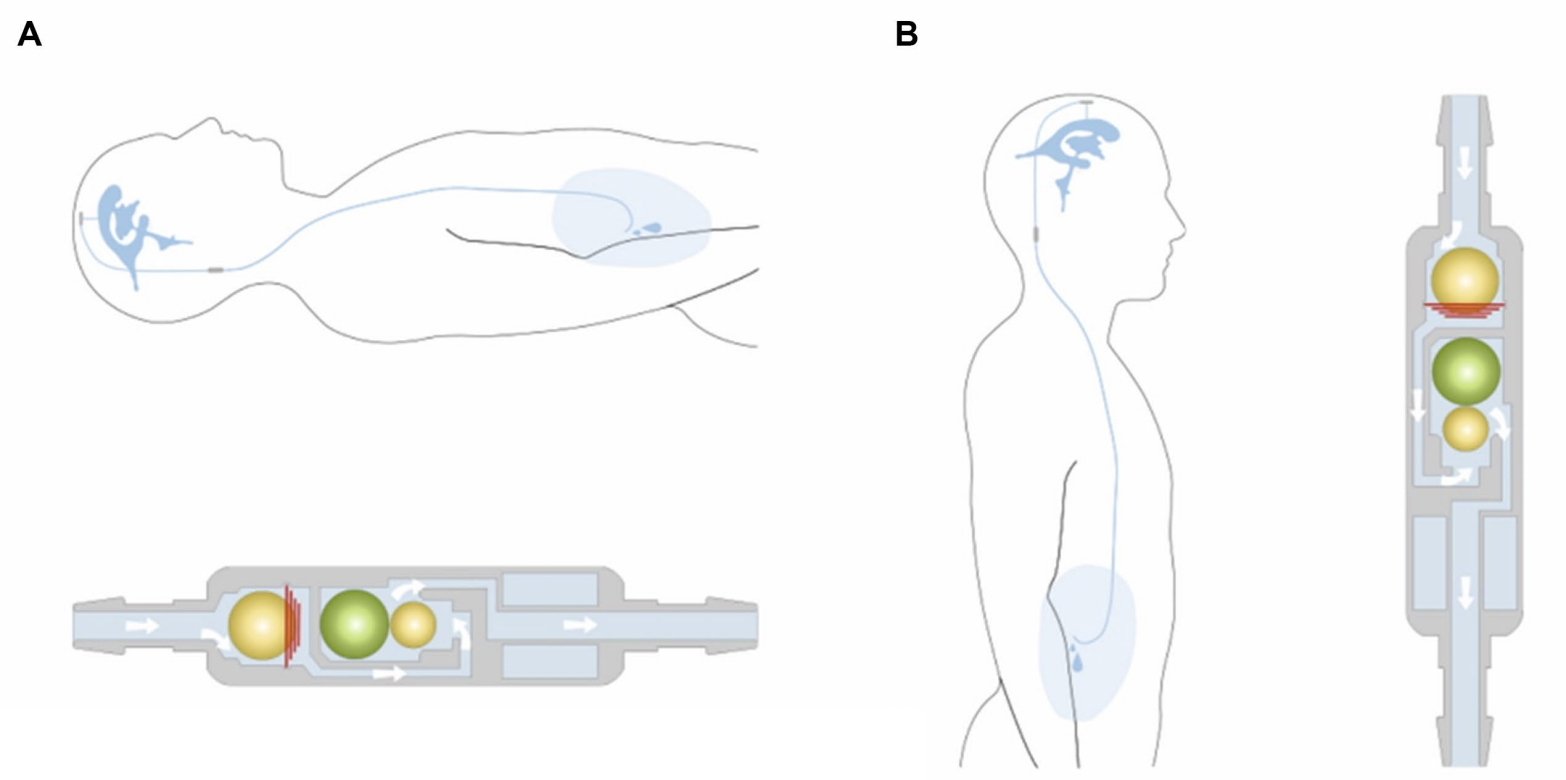
Example of a gravitational valve (GAV^®^; MIETHKE GmbH & Co. KG, Potsdam, Germany) that includes two valve chambers arranged in parallel. The first is a differential valve system with a prefixed opening pressure. In a horizontal position, this valve is the only one that resists flow. CSF flows without restriction through a second valve chamber. The gravitational unit starts working when the patient assumes an upright position. The postural changes move one or two balls from their original position to close the flow path in the second chamber. Permission granted by Christoph Miethke GmbH & Co. KG; www.miethke.com.

These differential valves are available in several prefixed pressure ratings. Valves with opening pressures between 0–5 cm of H_2_O are defined as low-pressure, 5–10 cm of H_2_O as medium-pressure, and 10–15 cm of H_2_O as high-pressure valves (2). The drainage rate of the shunt system is not only determined by the IVP, but also by the intraabdominal pressure and the hydrostatic pressure column in the catheter (13). In humans overdrainage can occur in the standing position, when the hydrostatic pressure in the vertical catheter causes a siphon effect (14). To avoid overshunting, gravitational valves are used in humans and dogs (10). As soon as the patient (human) moves into an upright position, the gravitational unit inside the valve (antisiphon device) closes and the total opening pressure of the valve is significantly increased (Figure 4).

An alternative strategy to avoid overshunting is the gradual selection of the optimal opening pressure, which is the highest setting that still allows for CSF drainage, and which is reflected by the patient’s clinical improvement in human neurosurgery. Such programmable shunt systems allow the opening pressure to be gradually altered non-invasively using a special magnetically activated mechanism. The opening pressure is gradually decreased until the clinical signs of the patient improve (15). Programmable shunt systems and the necessary equipment for external pressure adjustment are extremely costly, which limits their use in companion animals.

**Figure 5:**
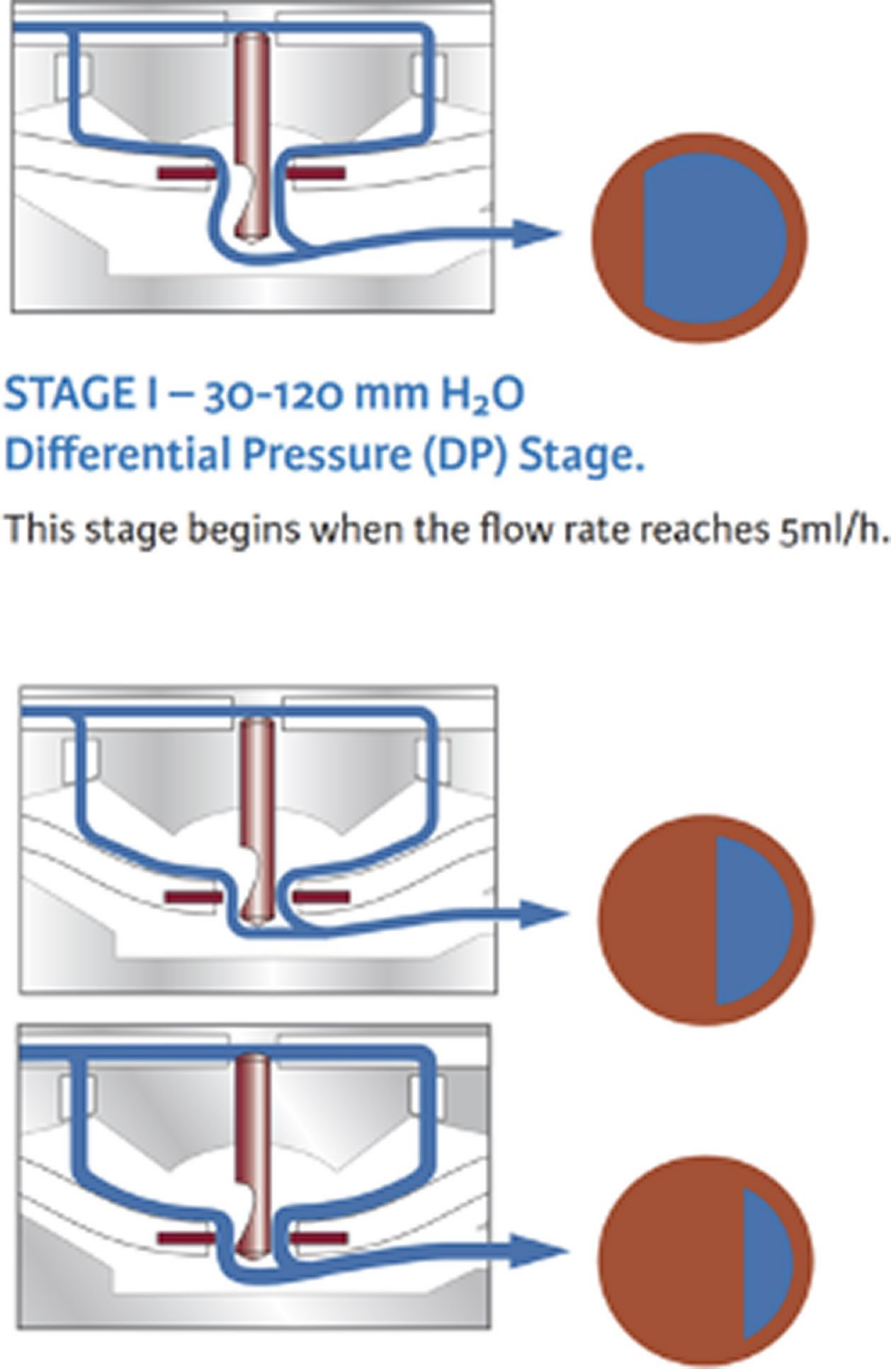
Example of a flow-regulating valve (Orbis Sigman valve; Integra neuroscience) that consists of a flow control rod that projects through an opening in a silicone membrane (flow orifice). The pin has a drop-shaped cut-out on its distal end that determines the diameter of the flow orifice and thereby the CSF volume flowing through the orifice. With increasing pressure, the membrane is deflected and creates a smaller orifice. Permission granted by Integra Life Science.

Flow-regulating shunt systems can avoid overshunting by limiting the total amount of CSF that is drained from the ventricles per hour. These valves increase the hydrodynamic resistance when CSF flow increases. Some examples include the SiphonGuard device (Codman) and the Orbis-Sigma OSV II valve (Integra Neuroscience) (Figure 5). The great advantage of flow-regulating devices is that they can be placed at any level of the tube and require no particular orientation (horizontal vs. vertical), which makes them useful for quadrupeds. They also avoid non-postural overdrainage caused by physical exertion, coughing, abdominal underpressure, etc. However, the minimal regulated flow volume of 5 mL per hour is adapted to a human CSF production rate and exceeds the canine or feline CSF production rate (13). This technical characteristic rather limits the benefit in dogs and cats.

### Indication for surgery

The diagnosis of congenital hydrocephalus is usually straightforward, as ventricular expansion is severe and causes craniofacial dysmorphia and doming of the calvaria in affected animals. In subadult or adult animals in which these skull changes might not be seen, the diagnosis of hydrocephalus may be difficult to establish, particularly in small brachycephalic dog breeds that tend to have relatively larger ventricles (ventriculomegaly) in comparison to mesocephalic dogs (16). It has been widely accepted that this increase in ventricular volume is not associated with clinical signs and VPS is not indicated (17–20). Morphological studies have shown, however, that dogs with ventriculomegaly have reduced cerebral white matter volumes and impaired periventricular perfusion, as in clinically relevant hydrocephalus (18, 21). Thus, ventriculomegaly should be seen as a form of chronic and potentially arrested hydrocephalus that may even show normal intraventricular pressure (18, 21). However, it is currently unclear as to whether ventriculomegaly is a progressive or non-progressive condition, and what the negative consequences of a slowly dilating ventricular system would be. There is no threshold level of ventricular volume that discriminates between internal hydrocephalus and ventriculomegaly, and even high grades of ventricular distension were diagnosed without the presence of clinical signs (16). It is therefore important to guarantee that the presented clinical signs are caused by ventricular distension and not by other brain diseases.

Small brachycephalic dogs are predisposed to meningoencephalitides of unknown origin (MUO) (22, 23), which might be present in addition to ventricular enlargement. MUOs might remain undetected, especially if CT is used as an imaging modality to diagnose internal hydrocephalus. Hence, it is thought that ventriculomegaly is sometimes misdiagnosed as relevant internal hydrocephalus and interpreted to be the cause of clinical signs in dogs that are affected by inflammatory/infectious disorders (18, 21). Amongst other signs, MUOs result in seizures, which are relatively rarely seen in animals with hydrocephalus (5). In cats with internal hydrocephalus, CNS infection with feline infectious peritonitis (FIP) virus must be ruled out. Among other consequences, the virus causes ependymitis and choroid plexitis, which both impair CSF flow, such as through the mesencephalic aqueduct and out of the lateral apertures (24, 25). Although ependymitis and choroid plexitis are often detectable in MRI (24), imaging findings may be absent (25). Neutrophilic pleocytosis in CSF has a higher sensitivity for detecting FIP but may also be absent in some cases. Surgery should be planned only in absence of MRI and CSF findings that indicate inflammatory changes and with a negative RT-PCR result for FIP antigen obtained from CSF (25, 26).

The most common clinical signs associated with internal hydrocephalus are visual impairment, obtundation, ataxia, behavioral changes (circling, aimless barking), and ventrolateral strabismus. Head tilt and nystagmus can be associated with distension of the fourth ventricle (10). In addition to the association with classical clinical signs, MRI signs indicative of increased IVP might be helpful to diagnose clinically relevant internal hydrocephalus (16, 27). The presence of tetraventricular hydrocephalus, periventricular edema, and signal void sign in the mesencephalic aqueduct are indicative of IVP >12 mm HG (normal range 6–12 mm HG) (27). However, animals with hydrocephalus can have variable IVPs. IVP can be elevated, but also within normal ranges, and even below normal physiological values (9). The absence of raised IVP does not imply that the animal will not benefit from shunting (9). In summary, the above-mentioned clinical signs and absence of inflammatory and other brain disease in association with ventricular distension are currently the best indicators that identify animals that will benefit from VPS.

### Presurgical considerations

Dogs and cats with congenital internal hydrocephalus that are presented at a very young age of 2–4 weeks carry a high risk of anesthesia even for diagnostic imaging (28, 29–31). From experience, it is recommended to perform MRI and surgery at an age of at least 6 to 8 weeks minimum. If surgery is performed at such a young age, considerations must be taken on how much growth will be expected (e.g., Chihuahua vs. Rhodesian Ridgeback) to place enough loops for the shunt to expand.

**Figure 6:**
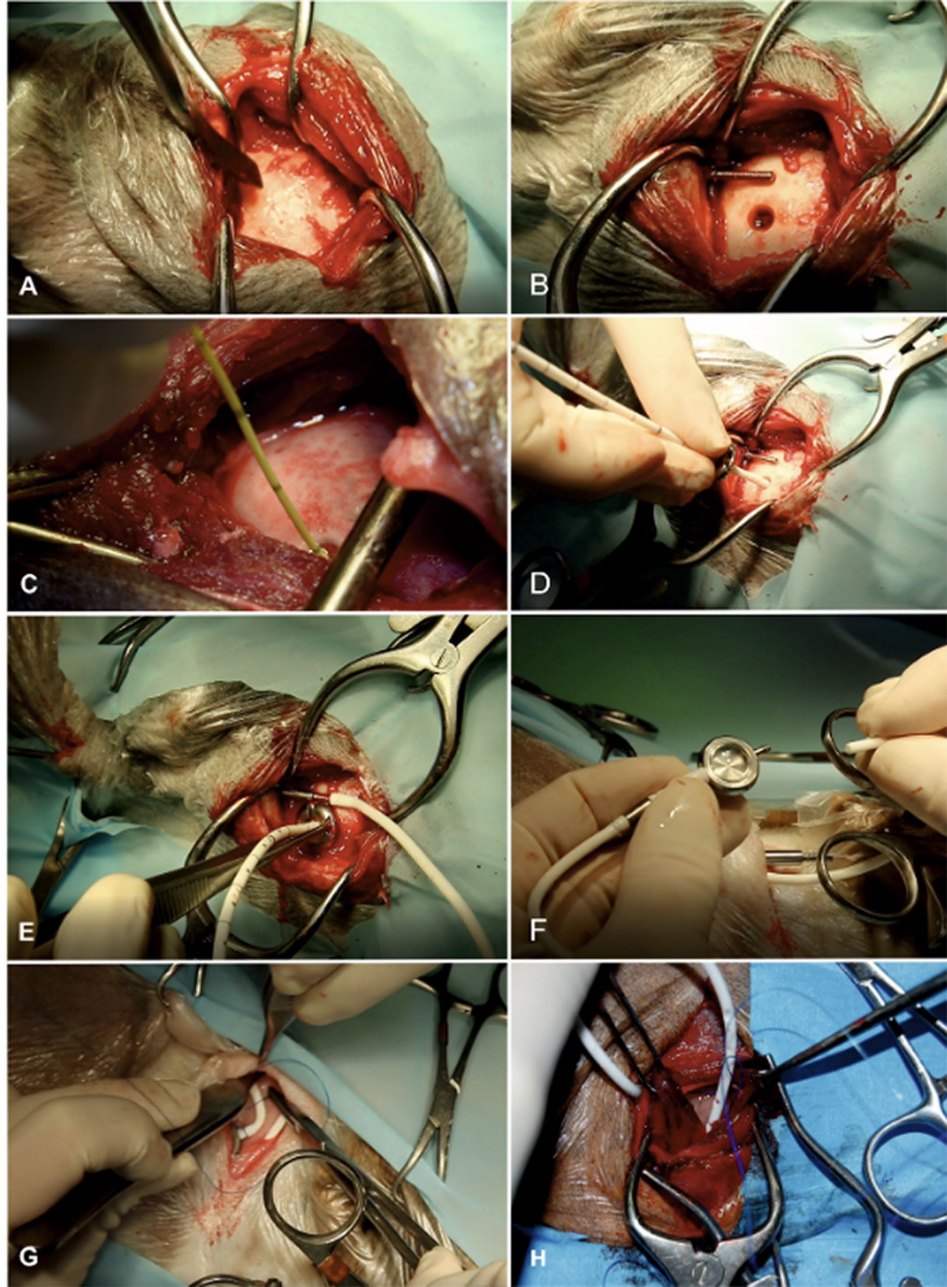
Surgical technique of ventriculo-peritoneal shunting (VPS). The hair on the lateral side of the cervical region, thorax, abdomen, and the whole head is clipped and aseptically prepared. Animals are placed in lateral recumbency. It is our aim to implant the shunt at the caudal horn to body transition where the dilation of the lateral ventricles is most pronounced, allowing for implantation with maximum depth. We also aim to position the shunt deflector underneath the masticatory muscles for protection and fixation. The most lateral point of the cranial vault (euryon) is palpated and the skull surface is exposed in this area **(A)**. A craniotomy hole is created in the skull with a pneumatic drill. The diameter must be adapted according to the diameter of the ventricular catheter. The dura is opened with a scalpel blade. Hemorrhage must be thoroughly controlled by bipolar cautery. A shunt passer is used to pass the distal end of the ventricular catheter underneath the masticatory muscles and then subcutaneously to the destination site in the lower dorsolateral cervical area. This needle must be placed before insertion of the ventricular catheter **(B)**. Intraventricular pressure is measured as a basis for the selection of an appropriate valve **(C)**. The cerebral cortex is then perforated with a stylet of a 20-gauge IV catheter. After punctuation, the ventricular catheter is inserted without resistance **(D)**. The tube is put in the deflector, which allows bending of the shunt without obliteration of its lumen. The distal end of the catheter is connected with the shunt passer and tunnelled under the muscles and skin towards the cervical incision **(E)**. The distal end of the ventricular catheter is connected to a CSF reservoir **(F)**. This dome is the centre for the S-shaped sling of the shunt tube. A narrow pocket is created craniodorsally and caudoventrally to the dome. After connection of the dome and the catheter parts, a semi-loop of the ventricular catheter is formed dorsally to the reservoir. The peritoneal catheter is connected and another semi loop is applied ventrally to the reservoir (or vice versa) **(G)**. These loops allow for stretching of the catheter system during head movements. The peritoneal catheter is again tunnelled under the skin towards the caudal aspect of the thoracic wall **(H)**. It is inserted in the peritoneal cavity via a small incision approximately 3 cm caudal to the last rib. Depending on the animal’s size, the inserted tube length should be between 5 and 10 cm. Several holes are incised into the distal part of the abdominal catheter to allow CSF drainage along a long section of the catheter and to avoid obstruction of the tip by the abdominal momentum. The catheter is attached to the abdominal wall by a Chinese finger-trap suture. Closure of the abdominal wall is routine.

Proper placement of the ventricular catheter at the largest point of ventricular enlargement is seeked (Figure 6). The ventricular catheter should be placed within the lateral ventricle with its tip ending at the level of the midline of the cerebral hemispheres to make sure a reconstitution of brain parenchyma is possible without obstruction of the catheter by the parenchyma. The depth of the aimed insertion length should therefore be measured on transverse MRI pictures before surgery.

As skull shapes vary among different brachy- and mesocephalic breeds, the ideal insertion point varies among individuals (32, 33). The aim is to place the ventricular catheter into the lateral ventricle but due to differences in skull shapes definite landmarks are hard to provide. Previous MRI might help to find the proper localization for shunt insertion and identification of some useful landmarks, e.g., temporal muscle lining, occipital protuberance, coronal sutures. Chihuahuas are often presented with internal hydrocephalus, but this breed is also common for large open fontanells and unossified sutures among the cranial vault (34, 35). This complicates proper shunt placement in these animals and might even make it impossible in rare cases.

Intraoperative IVP measurements are recommended to assess intraventricular pressure and choose an appropriate pressure valve system. Animals with an IVP of <5 mm Hg receive a differential pressure valve with an opening pressure of 5 cm H_2_O, in animals with an IVP between 6 and 12 mm Hg receive valves with an opening pressure of 10 cm H_2_O. In case of an IVP >13 mm Hg a 15 cm H_2_O valve is commonly used (10). The opportunity to measure IVP is not available in most facilities, therefore a neurological examination and MRI interpretation might help to identify animals with normal IVP and those with high IVP to choose an appropriate low (5 cm H_2_O) or high-pressure valve system (10–15 cm H_2_O). Obtundation, nystagmus, and head tilt are clinical signs associated with increased IVP in dogs, whereas the involvement of all 4 ventricles, the presence of periventricular edema, and a T2-signal void sign within the ventricular spaces are associated MRI signs (27).

Concurrent disease like otitis externa or dermatitis are contraindications for surgery and should be treated and resolved before VPS placement to avoid infections.

## Shunt-associated complications

### Obstruction

**Figure 7:**
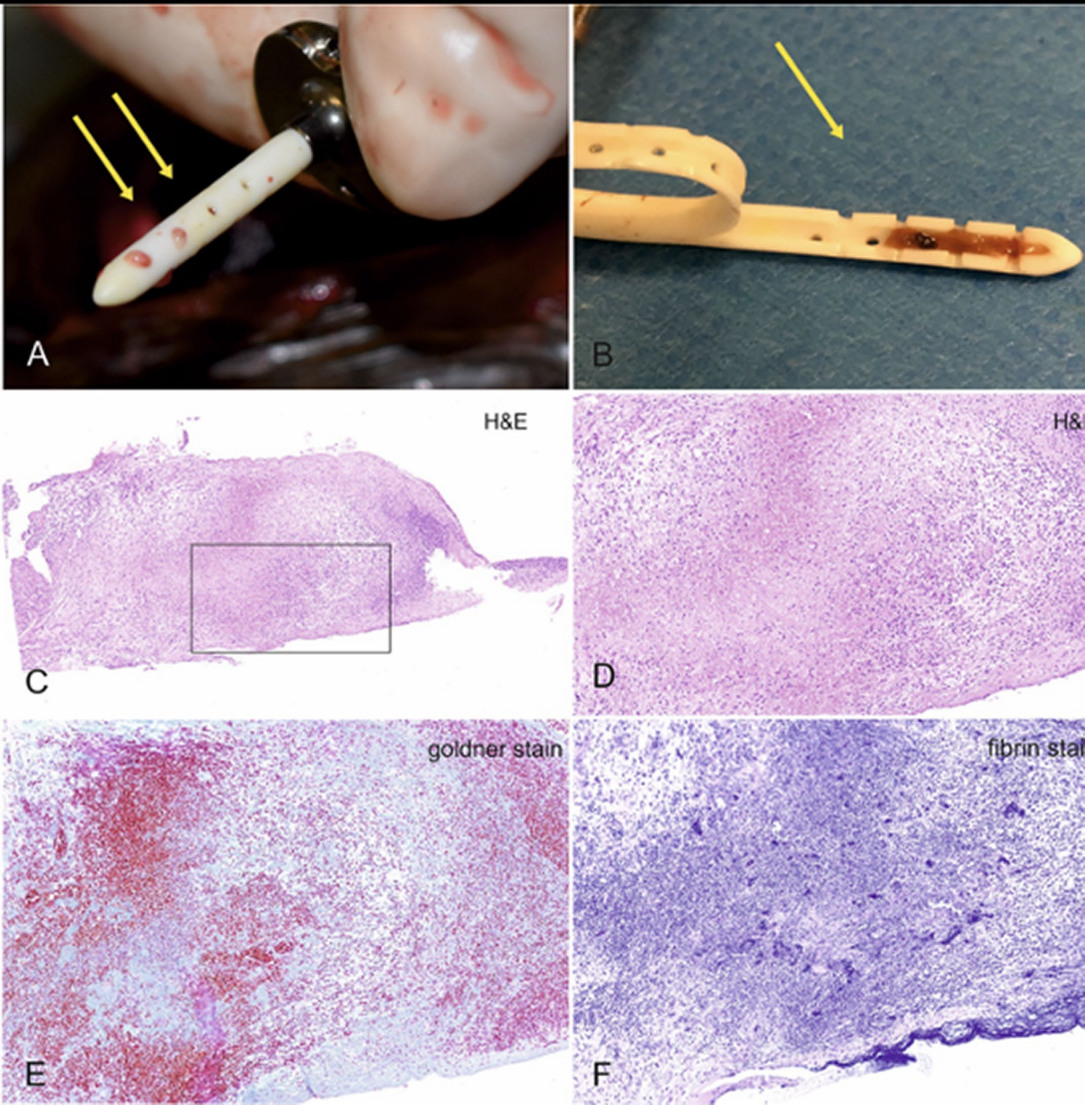
Typical appearance of a blocked shunt after removal from the ventricles **(A)**. After making a longitudinal cut through the catheter, blockage of the lumen within the first three drainage segments becomes obvious **(B)**. The last two segments were ensheathed by cerebral tissue and rendered useless. Histologic examination of the containing material revealed a mixture of granulation tissue with suppurative inflammation, erythrocytes, cellular debris, focal calcium deposits and siderophages, embedded in a collagen- and fibrin-rich stroma. **(C)** Overview, **(D)** 20× magnification, **(E)** Goldner stain for collagen, which is stained green-blue, and fibrin stain, which renders fibrin purple.

A shunt is designed to stay implanted for a lifetime. However, shunt obstruction continues to be a common problem and might require replacement of various components. The silicone tube is a foreign material that can result in an intraventricular or intraparenchymal inflammatory reaction (36) (Figure 7). CSF proteins can adhere to shunt surfaces and promote cellular attachment and deposition of debris. Obstructions can occur for various reasons and may be divided into obstructions resulting from cellular debris or kinking of the catheter. Obstruction from cellular debris or parenchymal cellular infiltration occurs in about 10% of VPS (8).

In humans, obstruction can either occur in the ventricular catheter, inside the shunt valve, or in the peritoneal catheter. Shunt flow studies are routinely conducted to localize the obstruction site. By injecting a small volume of contrast agent into the shunt reservoir, the flow of CSF through the catheters and valve can be verified (37, 38). In our cohort of dogs and cats, the main site of obstruction was the ventricular catheter. Obstruction of the peritoneal catheter occurred in only one dog in our cohort, and the valve was never obstructed.

Correct positioning of the ventricular catheter is crucial to avoid catheter obstruction. The drainage segments must project freely into the ventricular space. Contact with the cerebral parenchyma must be avoided, as this increases the likelihood of ventricular catheter occlusion due to ingrowth of inflammatory cells into the catheter lumen (39, 40). The cerebral parenchyma usually re-expands in dogs and cats after successful shunting, resulting in encasement of the catheter and subsequent mechanical obstruction (2). It is therefore important to insert the catheter as deep as possible into the ventricles. In humans, the ventricular catheter needs to be positioned as far as possible away from the choroid plexus, which tends to block the catheter (41). The occipital horn is the most common location for implantation (41). Data on the influence of the insertion site of the catheter on the occurrence of catheter occlusion in animals are not available. Based on our experience, the choroid plexus is not involved in catheter obstruction in animals and special strategies to avoid placement of the catheter tip near the choroid plexus are not made (see Figures 5–7).

In the postoperative phase, the reservoir should be repeatedly pressed to confirm the refilling and patency of the ventricular catheter. After discharge, owners can use the reservoir to check if the shunt is patent. After compression of the dome it becomes concave and should re-inflate quickly to its previous convex shape, indicating that the shunt is refilling with CSF from the ventricles. A lack of reinflation is indicated by the dome keeping a concave shape after compression and indicates proximal obstruction of the ventricular catheter. If animals are readmitted due to lack of reinflation or recurrence of neurologic signs, radiographs of the entire course of the shunt tube must be obtained to rule out dislocation of the ventricular catheter, kinks, or disconnections in the shunt system. Absence of CSF after puncturing of the reservoir is another clear sign of a blocked ventricular catheter. If the ventricular catheter is blocked it needs replacement. Flushing of the ventricular catheter is not effective. CSF or cellular material taken from the removed catheter and CSF taken directly from the ventricle should be submitted separately for bacterial examination (see below). Even if the bacterial examination is negative, the use of corticosteroids to reduce the inflammatory reaction against the shunt must only be considered with caution, especially as its benefits are not yet proven.

### Overshunting

**Figure 8:**
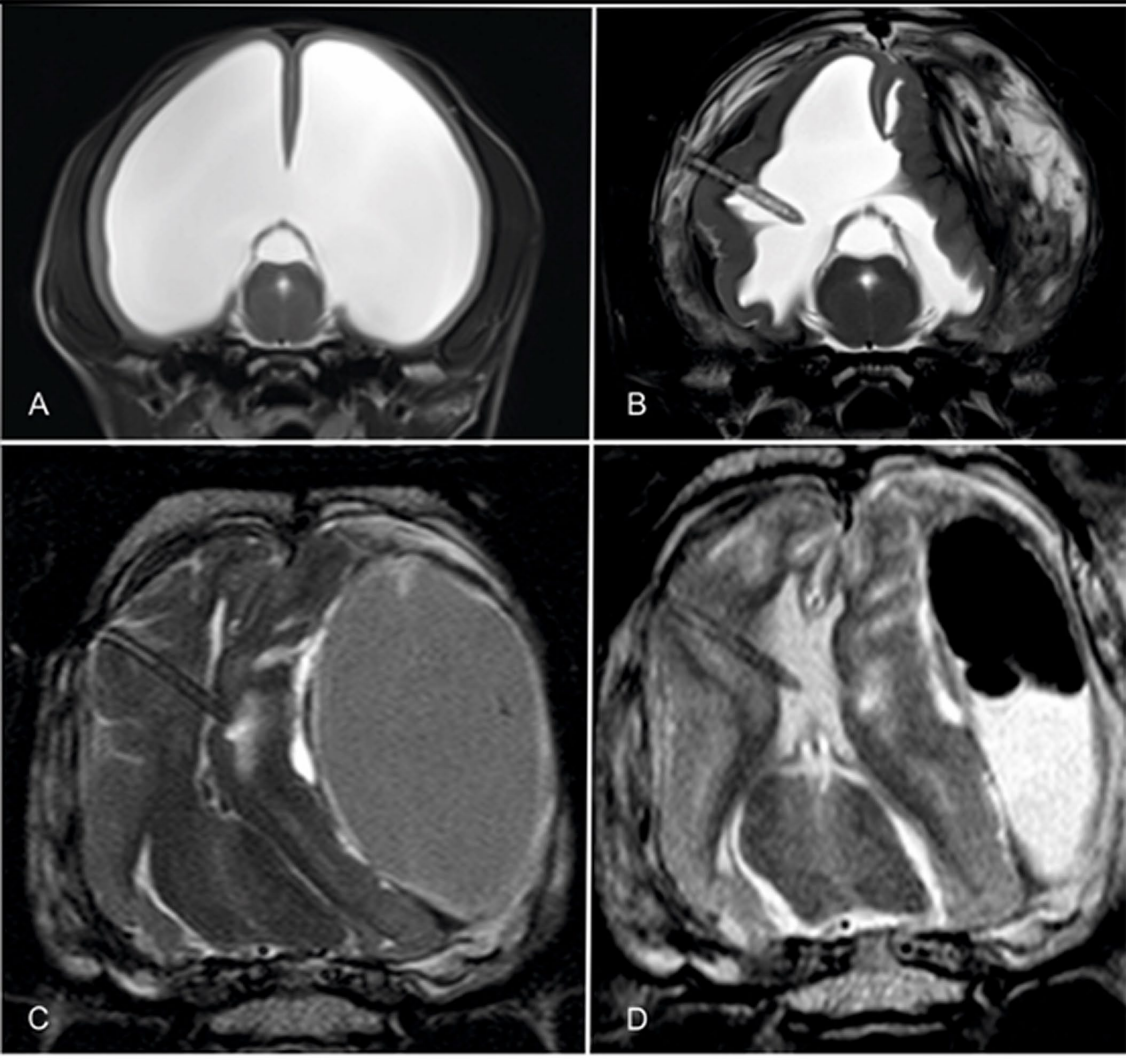
Transverse T2-weighted images through the brain of a hydrocephalic Chihuahua before **(A)** and after shunting and collapse of the right hemispheres and formation of an ipsilateral and contralateral hematoma **(B)**. The thickness of the hemisphere increased after shunting when the shunt was still working. Note the tissue that is being sucked towards the catheter tip. The ventral images show the brain of a mesocephalic mixed breed dog after implantation of a shunt. The ventricular space is totally diminished and the ventricular catheter is ensheathed with cerebral parenchyma **(C)**. After craniotomy and evacuation of the bulk of the hematoma, both the ventricles and the cerebral parenchyma partly re-expanded and the shunt continued draining CSF **(D)**.

Overshunting describes a condition in which too much CSF is drained out of the ventricle too quickly, resulting in intraventricular under-pressure and thus tensile stress on the brain tissue. This tensile stress pulls the surface of the cerebral cortex away from the cranial wall, causing small bridging veins to rupture. The consequence is a slowly expanding intra-arachnoidal hemorrhage (10). This accumulation of blood causes compression of the hemispheres and potentially hemispheric collapse (Figure 8). The prevalence of overshunting-related complications such as hemispheric collapse and subdural hematoma in animals was 18% in a recent study (10). Signs of ventricular collapse are peracute occurrence of neurological signs.

Hematoma formation can result in subtentorial and intraforaminal herniation of brain tissue. In severe cases, the hemisphere of one side invaginates into the contralateral ventricle, which is fatal. Biventricular hydrocephalus and high grades of ventricular distension (42) are risk factors for the development of hemispheric collapse (10). Craniotomy and evacuation of the hemorrhage are life-saving procedures, can restore ventricular drainage, and can also effectively improve clinical signs (10, 43).

The use of gravitational valves in animals to prevent overshunting is somewhat controversial. One of the unanswered questions is how gravity affects CSF drainage in quadrupeds. Due to differences in the body axis in humans and animals, the shunt tube runs relatively vertically in both the lying and standing positions. Based on the assumption that a sitting position increases hydrostatic pressure in the shunt tube, gravitational valves were used in hydrocephalic dogs, but could not effectively prevent overshunting (10).

### Other causes of shunt failure

Infection is the second most common cause of VPS malfunction in humans. Reported shunt infection rates range from 5 to 18%, with children under 1 year of age having the highest infection rate (44). It was suggested that the association of young age and infection may be caused by immaturity of the immune defence including the skin barrier, as well as delayed wound healing, and the etiology of intraventricular hemorrhage (44). Compared to humans, infection is a relatively rare source of complication in dogs and cats (4.1%) (8). As shunt infection usually results in obstruction, recurrence of neurologic signs is also observed. As mentioned above, shunt infection does not necessarily result in brain infection. Depression, lethargy, craniospinal pain and fever are only observed if infection spreads to brain tissues, and not in shunt infection alone. MRI is indicated to reveal signs of encephalitis, meningitis and ventriculitis that need to be treated aggressively (45). Bacterial cultures taken from the shunt are mostly negative (8). In our cohort, *Staphylococcus epididermidis* and *Staphylococcus pseudintermedius* were the most common bacteria in positive cultures. In both encephalitis and pure shunt infection, removal of the infected shunt is indicated. The use of antibiotic-impregnated shunt tubes in humans is very effective at preventing recurrent infection/obstruction but not for treating encephalitis (Bactiseal^®^ Integra Lifescience; XABO^®^ MIETHKE) (46), as the antibiotic is only released into the shunt lumen. It is important to note that use of antibiotic agents in the tube (combination of rifampicin and clindamycin) may be restricted by European regulations governing antibiotic use in veterinary medicine.

**Figure 9:**
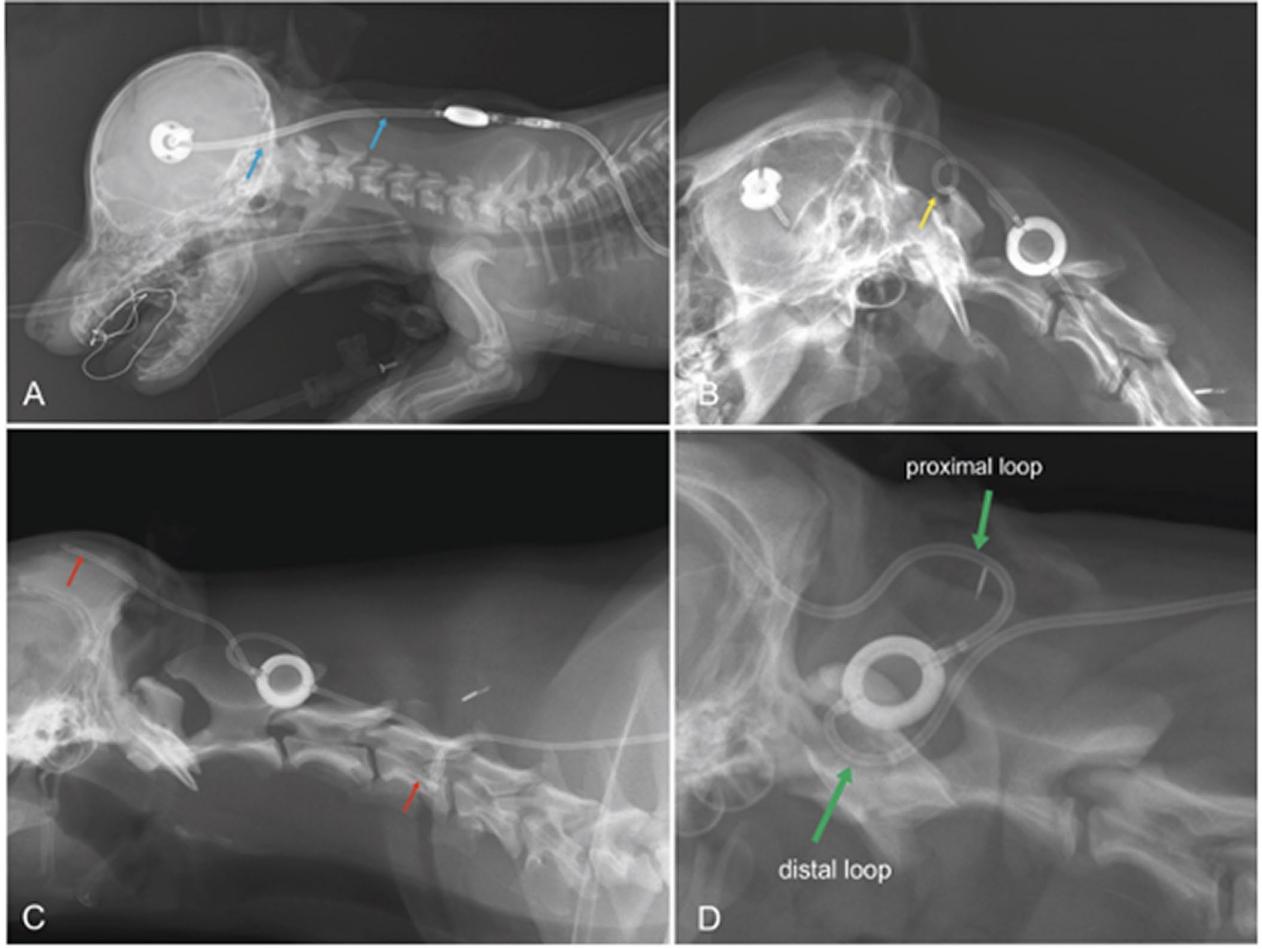
Laterolateral radiographs displaying the postoperative condition of dogs after VPS. In a Pinscher dog **(A)**, the distal end of the ventricular catheter is too straight (blue arrows). A sudden turn of the head and neck away from the implants will likely pull the catheter out of the cerebral ventricles. A tight expansion loop, as seen in **B** (boxer dog, yellow arrow), will likely result in transient kinking of the shunt on the ventral aspect of the loop, which might be permanent after the formation of scar tissue. The loop in **C** (Labrador retriever) was also not effective at preventing catheter migration (red arrow). In addition, there is a kink at the level of C5 (red arrow). A proximal and a distal half-loop is recommended (**D**; green arrows), as these can extend during stretching of the neck and there is no risk for coiling of the tube in the subcutaneous tissue (**D** Samoyed).

Dislocation of the ventricular catheter and kinking of the subcutaneous tube are other rare complications. Occurrence of shunt dislocation can be reduced if expansion loops are placed underneath the skin, especially along the course of the tube that runs between ventricular catheter and reservoir. On the other hand, these loops are predisposed to kinking if not properly applied (Figure 9). However, especially in large dogs with long movable necks, or in puppies that underwent surgery at a very young age, shunt dislocation can be a serious and perennial problem.

### Postoperative development and follow-up care

In primary/idiopathic hydrocephalus, neurologic dysfunction results from stretching of the periventricular white matter, as well as from impaired perfusion by periventricular blood vessels and impaired bulk flow of CSF within the brain tissue, leading to interstitial edema (18, 47, 48). VPS has the potential to reduce the ventricular dimensions, and allows parenchymal reconstitution. Based on our experience, signs of obtundation resolve very quickly within 12–24 h after surgery. Ataxia and cerebello-vestibular signs, on the other hand, take longer to improve (3–5 days). The presence of cerebello-vestibular signs in the first 2–3 weeks after surgery may raise the suspicion of a persistently dilated fourth ventricle, and a repeat CT/MRI is indicated. Based on our experience, the fourth ventricle is often not sufficiently drained, even if the dimensions of the lateral and third ventricle are decreased. The reason for this phenomenon is uncertain. Suboccipital craniectomy and incision of the caudal medullary velum can effectively remove CSF from the fourth ventricle, which results in permanent clinical improvement (unpublished data).

Visual deficits are in general hard to assess in animals and improvement of visual capabilities is likewise very difficult to determine. If destruction of the optic radiation is seen on preoperative MRI, central blindness will likely be permanent (2).

A regular follow-up neurological examination and repeat MRI is recommended after 3 months to evaluate clinical improvement, shunt function and possible reconstitution of brain parenchyma. If the animal has improved clinical signs and remains clinically unremarkable, there is actually no rationale for further regular follow-up. If the animal has not improved, it must be scheduled for re-examination, in which shunt patency must be checked (see below). If the shunt is patent and clinical signs have not improved, MRI should be considered. If ventricular volume has not decreased, implantation of a valve with a lower opening pressure should be considered. If ventricular size has decreased, and there is no clinical improvement, the lack of improvement must unfortunately be attributed to brain damage/atrophy that might persist after a decrease in ventricular volume (2).

### Medical therapy

Medical therapy with prednisone, omeprazole, and acetazolamide to decrease CSF production by alteration of ion concentration and passive water current into the CSF space has been previously described for dogs and cats with congential internal hydrocephalus (48–50). But several studies describe no or just a limited effect of those drugs (6, 51–53).

Omeprazole was proven to reduce CSF production in experimental studies in dogs and rabbits if administered intravenously or intrathecally (54, 55). Oral administration (0.5–1.5 mg/kg) has therefore been proposed for long-term treatment of syringomyelia and internal hydrocephalus (1, 48, 49). However, oral dosages of 1 mg/kg are proven to be ineffective to lower CSF production in dogs (53).

The efficacy of glucocorticoids on CSF production has never been investigated and there is no evidence of efficacy for the treatment of internal hydrocephalus or syringomyelia in the current literature. Some studies however suggest their use for treatment of internal hydrocephalus and syringomyelia (48–50), but the effect is probably limited to the reduction of edema and inflammatory reactions rather than lowering CSF production (56).

Acetazolamide is the only drug with proven effect on CSF production by oral administration route in dogs and other species (52, 57–59). Nonetheless, acetazolamide is proven to be ineffective to reduce clinical sings and ventricular volume in a long-term follow up (6). Increased production of osmogenic ions from the ependyma and choroid plexus and an upregulation of acetazolamide-resistant isozymes occur after long-term use of acetazolamide. This could be the reason for the time limited effect on CSF production which lasts about 2-6 weeks (51, 52). It, however, may be used to lower CSF production for a few weeks, until a VPS surgery can be performed.

### Alternative surgical procedures

Ventriculo-atrial shunts divert excess CSF into the internal jugular vein and further into the right atrium. An advantage of the procedure is the permanent intravenous pressure that resists CSF outflow if IVP falls below intravenous pressure, avoiding overshunting without a shunt valve. Ventriculosinusal shunts are other forms of vascular ventricular drainages that place the distal catheter in the sagittal-, or transverse sinus. The major disadvantage of these techniques is the risk of venous thrombosis and sepsis causing a high mortality (60). A plethora of alternative nonvascular ventricular drainages have been described including ventriculopleural shunt, ventriculo-gallbladder shunt, ventriculoureteral shunt, as well as drainage into the thoracic duct, or spinal epidural space. These techniques are used to avoid complications specific to VPS (i.e., intraperitoneal adherence, visceral perforations etc.) (61). Lumboperitoneal shunts (LPS) can be used to treat communicating hydrocephalus. VPS and LPS are equally effective to improve clinical signs in humans. The main advantage of the technique is that it is less invasive, avoids brain penetration and potential associated complications (parenchymal damage, subdural hemorrhage) (62). There is currently no experience with these techniques in veterinary medicine.

Procedures that do not use shunt systems are also scarcely described in hydrocephalic animals. Experience with ventriculo-subarachnoid fistulas, in which a connection between the ventricles and the subarachnoid space is produced, are limited to only few descriptions and their longterm outcome is uncertain (63).

Endoscopic third ventriclulostomy is a procedure mostly used to treat obstructive hydrocephalus in children (64). The procedure aims to create a passage between the third ventricle and the prepontine cistern. Again, there are no reports describing ETV in animals. When, or if, more evidence or experience is documented, these other techniques might be considered for the treatment of hydrocephalic animals in the future.

## Conclusion

Surgical treatments for internal hydrocephalus continue to evolve. Refining patient selection and tailoring appropriate valves to the individual IVP is an important step in improving treatment. Proper placement of the ventricular catheter reduces the risk of catheter obstruction. Shunt disconnection and overshunting continue to be serious complications after VPS.

## Author contributions

MS: Conceptualization, Data curation, Formal analysis, Investigation, Methodology, Project administration, Resources, Validation, Visualization, Writing – original draft, Writing – review & editing. DF: Conceptualization, Data curation, Formal analysis, Investigation, Methodology, Project administration, Resources, Validation, Writing – original draft, Writing – review & editing.
